# A two‐step infection model: From SARS‐CoV‐2 entry and trafficking to egress and spread

**DOI:** 10.1002/mco2.295

**Published:** 2023-05-31

**Authors:** Liting You, Binwu Ying

**Affiliations:** ^1^ Department of Laboratory Medicine West China Hospital Sichuan University Chengdu Sichuan China

1

In a recent study in *Cell*, Wu et al.^1^ reported a new model deciphering the SARS‐CoV‐2 infection mechanism in which the virus binds to ACE2 on ciliated epithelial cells, accesses the cell body, egresses from rearranged microvilli, and spreads via mucociliary transport (MCT) (Figure 1A). They tracked the detailed infection processes and demonstrated that motile cilia and microvillar reprogramming are critical for virus replication. In addition, their data explain why Omicron spread more efficiently than previous variants and argue for targeting cilia or microvilli to impede virus entry or spreading. In this article, we will highlight the infection model step by step.

**FIGURE 1:**
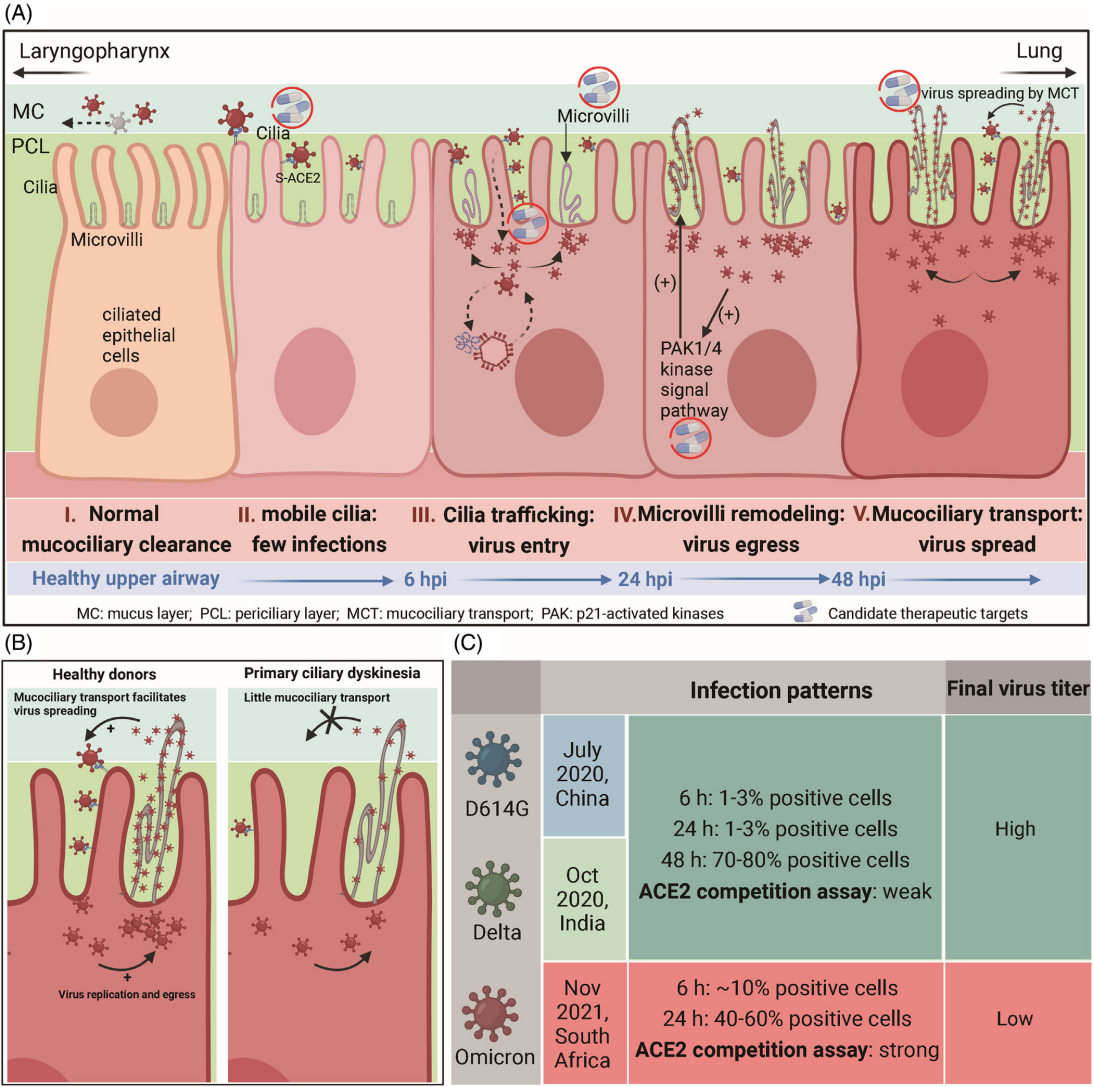
Model of SARS‐CoV‐2 infection patterns and mechanisms. (A) In healthy individuals, mucociliary clearance is an important link in airway mucosal defense. Mobile cilia in the tip of nasal epithelial cells can sweep pathogens for elimination. On the apical cell membrane of ciliated columnar cells, short and thin microvilli exist and their function has not been fully elucidated (I). Therefore, only a few epithelial cells are infected when SARS‐CoV‐2 enters the upper respiratory tract (II). Within the next 24 h postinfection, ciliary trafficking facilitates virus movement to the cell body, and then viruses replicate by utilizing materials of host cells (III). SARS‐CoV‐2 activates the p21‐activated kinases 1 and 4 (PAK1/4) signaling pathway to regulate microvilli remodeling (apically extended and highly branched) and is shed from the remodeled microvillus (IV). As mucociliary transport (MCT), viruses spread and infect neighboring cells, repeating the above processes (V). Mobile cilia, microvilli, protein trafficking, PAK1/4, and MCT are candidate therapeutic targets to prevent infection by SARS‐CoV‐2 and other respiratory viruses. (B) Primary ciliary dyskinesia (PCD) patients have poorer MCT function than healthy donors. Thus, SARS‐CoV‐2 spreading is limited at later stages of infection, and PCD patients have little risk of infection or transmission or severe pneumonia. (C) Different infection patterns of D16G, Delta, and Omicron. (Figure 1 was created in Biorender.com.)

2

Mucociliary clearance comprises one of the most important links in airway mucosal defense. Mobile cilia and microvilli in the tip of nasal epithelial cells can sweep bacteria and virus particles to the laryngopharynx for elimination. However, cilia and microvilli, originally defensive structures, may be hijacked by SARS‐CoV‐2 to facilitate virus infection. By infecting human nasal epithelial organoids with virus at the air–liquid interface (ALI) and observing using multiple imaging techniques, Wu et al. found that SARS‐CoV‐2 attaches to ciliary ACE2 receptors to facilitate its entry. Depleting cilia while maintaining ACE2 receptor expression impedes SARS‐CoV‐2. These data suggest a virus entry model in which ciliary beating and retrograde trafficking are two key factors. In a previous study of ACE2 subcellular localization, Wu et al.^2^ first reported that ACE2 is robustly expressed in airway motile cilia. Almost 80% of human airway epithelial cells are covered with abundant cilia (50–200 cilia per cell) that are beating in coordination, which provides a large surface area for SARS‐CoV‐2 binding and entry. After SARS‐CoV‐2 binds to ACE2, protein trafficking in cilia facilitates virus movement from the top to the bottom of epithelial cells. Since the detailed mechanisms remain unclear, Wu et al. proposed the binding model and transport model for virus entry to be identified in the future. Their most prominent contribution was to clarify the importance of ciliary beating and retrograde trafficking in the process of SARS‐CoV‐2 infection and to extend this theory to other respiratory viruses. More significantly, excellent therapeutic effects and no apparent toxicity were observed in the cilia‐depleted or trafficking‐blocked experiments.

3

During the life cycle of infected epithelial cells, SARS‐CoV‐2 replicates genomic RNA, expresses viral proteins to produce complete viral particles and is then released. However, the detailed information and in‐depth mechanisms underlying the process remain unclear. Wu et al. first revealed microvilli structure is a crucial element in virus egress process. Scanning electron microscopy imaging in their earlier report has shown that massive SARS‐CoV‐2 virions are shed from microvilli 24 h postinfection, although the interaction between the virus and microvilli has not been further explored before.^3^ Excitingly in this study, Wu et al. discovered that SARS‐CoV‐2 virions regulate microvillus remodeling to facilitate virus egress and deciphered the underlying mechanisms. Morphologically, microvilli remodeling was characterized by a high degree of bifurcation and longitudinal extension. In 2D infection models (Caco‐2 and Vero cells), SARS‐CoV‐2 induced filopodia protrusion containing virions. In human nasal epithelial 3D organoids and infected airway tissues, massive SARS‐CoV‐2 virions are colocalized with microvilli and induce remodeling of morphology and structure, from stubby and dome‐like to tall and highly branched. Mechanistically, phosphoproteomic analysis and inhibition assays revealed that SARS‐CoV‐2 regulates microvilli molecular reprogramming by the p21‐activated kinase (PAK) 1/4 signaling pathway and identified several promising downstream targets.

4

Shedding from the microvilli filled with virions, SARS‐CoV‐2 particles traffic into the flowing mucus layer. A previous study reported that surface mucus works to eliminate foreign pathogens via MCT and that SARS‐CoV‐2 transfers through cell–cell contact or cell–cell fusion by the spike glycoprotein.^4^ However, Wu et al. did not observe significant virion aggregation in cell‒cell contact and assumed that transmission through cell–cell contact may not be a major pathway. By tracking and comparing the infection status of nasal epithelial cells from healthy donors and primary ciliary dyskinesia (PCD) patients with little MCT, they observed that the virus load was similar at 24 h postinfection, while at 48 h, PCD patients had fewer infected cells than healthy control (Figure 1B), suggesting that the widespread transmission of SARS‐CoV‐2 after entry into the airways depends on MCT. PCD patients have no increased risk of SARS‐CoV‐2 infection; further, they seem to show a lower risk of progression to severe disease and transmission risk in the clinic. In other words, healthy MCT in turn contributes to the virus spread. Like that, whether a similar pattern holds for the transmission of other respiratory viruses needs more evidence.

In addition, Wu et al. provided a rational explanation for the high transmissibility of the Omicron variant based on their model for virus entry and egress. Omicron variant, rapidly becoming the dominant variant upon first report in November 2021, has abundant alterations in the receptor‐binding domain of spike proteins and is constantly evolving, from BA.1 to BA.5. They identified the temporal and spatial infection patterns of different variants (D16G, Delta and Omicron) (Figure 1C) and compared the interaction fitness between their spike proteins and ACE2 receptors. These data collectively illustrated that Omicron spike proteins are more likely and stronger to bind to the receptors than that of other variants and replicate faster and much earlier. Targeting mobile cilia and microvilli can impede infections, suggesting that Omicron also follows the two‐step infection model. As for the other intriguing phenomenon that the virus load of Omicron infection after 48 h is lower than that of D16G and Delta, there are currently no validations or explanations to be declared yet according to their infection model. An additional point to emphasize is the SARS‐CoV‐2 infection models used in this study, including 2D cell infection model, ALI cultured organoid model, as well as infected airway tissues. 2D cell infection model differed greatly both in microenvironment cell composition and in vivo infection pattern. For example, SARS‐CoV‐2 virions rapidly spreads in 2D cells without an evident delay. ALI airway organoid model could mimic the morphology and function of normal human airway; however, many nuances exist between SARS‐CoV‐2 infection organoid and infected airway tissues. Prominently, ALI cultured organoid models has an evident kinetic delay (24–48 h) compared with tissue culture models. These discrepancies may affect the reliability of the conclusion and the possibility of clinical translation.

In summary, this study deciphered the SARS‐CoV‐2 infection mechanism and pinpointed the infection routes for entry, egress and spread, which is also followed by other respiratory viruses. In this process, cilia and microvilli are vitally important structures; the former facilitates early‐stage infection, and the latter modulates later‐stage infection. Accordingly, topical and transient inhibitors of these targets used in the upper airway may impede virus infection in different stages with few adverse effects, which provides new strategies for the clinical prevention of SARS‐CoV‐2. However, another important question to be identified is whether blocking these targets is efficient for SARS‐CoV‐2 patients in which viruses have replicated widely and immune inflammation has been ignited. This two‐step model may not be the only pathway for viruses and may represent one of the complex mechanisms in the relatively early stage of infection. The compensation function of human multidimensional lungs and immune activity should be taken into account.

## AUTHOR CONTRIBUTION

L. Y. wrote the manuscript draft and drew the figure. B. Y. revised and proofread the manuscript. All authors have read and approved the article.

## ETHICS STATEMENT

Not applicable.

## CONFLICT OF INTEREST STATEMENT

The authors declare no conflict of interests.

## Data Availability

Not applicable.

## References

[1] Wu CT , Lidsky PV , Xiao Y et al. SARS‐CoV‐2 replication in airway epithelia requires motile cilia and microvillar reprogramming. Cell 2023;186, 112–130 e120. 36580912 10.1016/j.cell.2022.11.030 PMC9715480

[2] Lee IT , Nakayama T , Wu CT et al. ACE2 localizes to the respiratory cilia and is not increased by ACE inhibitors or ARBs. Nat Commun 2020;11, 5453. 33116139 10.1038/s41467-020-19145-6 PMC7595232

[3] Morrison CB , Edwards CE , Shaffer KM et al. SARS‐CoV‐2 infection of airway cells causes intense viral and cell shedding, two spreading mechanisms affected by IL‐13. Proc Natl Acad Sci USA 2022;119, e2119680119. 35353667 10.1073/pnas.2119680119 PMC9169748

[4] Zeng C , Evans JP , King T et al. SARS‐CoV‐2 spreads through cell‐to‐cell transmission. Proc Natl Acad Sci USA 2022;119. 10.1073/pnas.2111400119 PMC8740724 34937699

